# Two Complete Mitochondrial Genomes of Mileewinae (Hemiptera: Cicadellidae) and a Phylogenetic Analysis

**DOI:** 10.3390/insects12080668

**Published:** 2021-07-22

**Authors:** Tinghao Yu, Yalin Zhang

**Affiliations:** Key Laboratory of Plant Protection Resources and Pest Management, Ministry of Education, Entomological Museum, College of Plant Protection, Northwest A&F University, Yangling, Xianyang 712100, China; 18404965218@163.com

**Keywords:** Auchenorrhyncha, leafhopper, mitogenome, phylogeny

## Abstract

**Simple Summary:**

Mileewinae is a small subfamily of Cicadellidae containing about 160 described species, extensively distributed in the Oriental, Ethiopian and Neotropical regions. Some species are potential pests in agriculture and forestry. The classification of this group has been unstable over the past few decades. Currently, some controversies remain on the monophyly of Mileewinae and phylogenetic relationships of Mileewinae with other subfamilies. To provide further evidence toward answering these questions, two newly completed mitochondrial genomes of Mileewinae species (*Mileewa rufivena* and *Ujna puerana*) have been sequenced and analyzed. Results show these two mitochondrial genomes have quite similar structures and features. In phylogenetic analyses, Mileewinae formed a monophyletic group in Cicadellidae in all trees derived from maximum likelihood (ML) and Bayesian inference (BI) methods. In addition, Mileewinae has a closer phylogenetic relationship with Typhlocybinae compared to the Cicadellinae.

**Abstract:**

More studies are using mitochondrial genomes of insects to explore the sequence variability, evolutionary traits, monophyly of groups and phylogenetic relationships. Controversies remain on the classification of the Mileewinae and the phylogenetic relationships between Mileewinae and other subfamilies remain ambiguous. In this study, we present two newly completed mitogenomes of Mileewinae (*Mileewa rufivena* Cai and Kuoh 1997 and *Ujna puerana* Yang and Meng 2010) and conduct comparative mitogenomic analyses based on several different factors. These species have quite similar features, including their nucleotide content, codon usage of protein genes and the secondary structure of tRNA. Gene arrangement is identical and conserved, the same as the putative ancestral pattern of insects. All protein-coding genes of *U. puerana* began with the start codon ATN, while 5 *Mileewa* species had the abnormal initiation codon TTG in *ND5* and *ATP8*. Moreover, *M. rufivena* had an intergenic spacer of 17 bp that could not be found in other mileewine species. Phylogenetic analysis based on three datasets (PCG123, PCG12 and AA) with two methods (maximum likelihood and Bayesian inference) recovered the Mileewinae as a monophyletic group with strong support values. All results in our study indicate that Mileewinae has a closer phylogenetic relationship to Typhlocybinae compared to Cicadellinae. Additionally, six species within Mileewini revealed the relationship (*U. puerana* + (*M. ponta* + (*M. rufivena* + *M. alara*) + (*M. albovittata* + *M. margheritae*))) in most of our phylogenetic trees. These results contribute to the study of the taxonomic status and phylogenetic relationships of Mileewinae.

## 1. Introduction

The mitochondrion has its own genes to control the cell cycle and cell growth in eukaryotic cells [1]. This self-governed DNA encodes several proteins and RNAs for formation of a functional mitochondrion. In recent years, with the progress of next-generation sequencing technologies, a mounting number of insect mitochondrial genomes have been sequenced and reported. Generally, insect mitochondrial genomes are a small (14–20 kb), circular, closed DNA molecule that covers four broad categories: protein-coding genes (PCGs), RNAs (tRNAs), ribosomal RNA genes (rRNAs) and a putative control region (CR) [2]. The whole mitogenome can provide a full set of genome-level characters, including the base composition, codon diversity and usage, secondary structures of tRNA and the function of a non-coding region. Due to the advantages of small size, strict maternal inheritance, simple genetic structure, fast evolutionary rate and very high copy numbers, the mitogenomes can be easily obtained and widely used in studies of species delimitation, phylogenetics, evolution and biogeography [3,4,5].

Mileewinae is a small subfamily of Cicadellidae containing about 160 described species, extensively distributed in the Oriental, Ethiopian and Neotropical regions [6]. Most species of this subfamily inhabit wet tropical rainforests and appear in greatest diversity and abundance in cloud forests of mountainous areas [7]. Morphologically, species of mileewine are small-to-medium-sized (3.5–7.0 mm), slender, black or dark brown and generally have a reduced clavus and transparent patches on their forewings [8]. Some species in this group (*M. branchiuma*, *M. margheritae*) can damage wormwood, bamboo and other crops and are potential pests of agriculture and forestry. Mileewini is the largest and most widespread tribe of Mileewinae, which has over 90 species belonging to four genera: *Mileewa* Distant 1908, *Ujna* Distant 1908, *Amahuaka* Melichar 1926 and *Processina* Yang, Deitz and Li 2005 [6].

Classification of this group has been unstable over the past few decades, and previous scholars have held disparate views. Mileewini was established by Evans (1947) and placed it in the Cicadellinae (as “Tettigellinae”) [9]. Young (1965) transferred this tribe from Cicadellinae to the subfamily Typhlocybinae [10]. However, this placement by Young was doubted by Mahmood (1967), and he moved this tribe back into Cicadellinae in his later related work [11]. Young (1968) first elevated Mileewinae to status as a separate subfamily, and this status has been accepted by several subsequent authors. Dietrich (2005) also accepted this perspective in his taxonomic study of the Cicadellidae family [12,13]. Then, Dietrich (2011) redescribed this group based on phylogenetic analyses of both molecular and morphological data, suggesting Mileewini and another three tribes (Makilingiini, Tinteromini and Tungurahualini) represented a monophyletic group, meanwhile treating Mileewinae as tribes of a single subfamily [7]. However, there are still many different opinions about the classification of Mileewinae, and the relationships among Cicadellinae, Typhlocybinae and Mileewinae have not yet been stated explicitly. In previous studies, three complete (*M. albovittata*, *M. margheritae* and *M. ponta*) mitogenomes and a partial mitogenome (*M. alara*) of *Mileewa* have been reported [14,15,16,17]. Analysis based on these mitochondria supported that Mileewinae was monophyletic, but it did not analyze the relationships between Mileewinae and other subfamilies.

Here, we present two new, complete mitogenomes of Mileewinae (*Mileewa rufivena* Cai and Kuoh, 1997 and *Ujna puerana* Yang and Meng, 2010) and provide comparative mitogenomic analyses of six Mileewinae species from genome size, nucleotide composition, codon usage, tRNA secondary structure, gene overlaps and intergenic spacers, evolutionary rate, and A + T-control region. We conducted phylogenetic analysis using maximum likelihood (ML) and Bayesian inference (BI) methods. The aims of our study were to: (1) explore the sequence variability and evolutionary traits of the Mileewinae mitogenomes; (2) test the monophyly of the Mileewinae; (3) investigate the relationships between Mileewinae and other subfamilies; and (4) explore the relationships among the species within Mileewini.

## 2. Materials and Methods

### 2.1. Sample Collection and DNA Extraction

Specimens in our study were collected in 2018 from China (Table S1). Specimens were initially preserved in anhydrous ethanol and then stored at −80 °C in the laboratory. We identified our specimens accurately based on morphological characters [18] and the gene fragment of cytochrome oxidase subunit 1 (standard mitochondrial *COX1* barcode) in GenBank.

The entire DNA genome was extracted from abdominal tissues by using the Biospin Insect Genomic DNA Extraction Kit (BioFlux, Hangzhou, China) following the manufacturer’s instructions. The genomic DNA was then stored at −20 °C, and vouchers were deposited in the Entomological Museum of Northwest A&F University (NWAFU), Yangling, China.

### 2.2. Mitogenomes Sequencing, Assembly and Annotation

Complete mitogenomes of Mileewinae were generated using the next-generation sequencing (methodology of the PE150 by Illumina HiSeq™ Xten platform; Novogene Technologies, Beijing, China) and PCR amplification. Amplified nucleotide sequences of NADH dehydrogenase 2 (*ND2*) were used to identify the vacant sites (compared with reference mitogenomes) that appear in the results of next-generation sequencing and make up for the gap. We used the universal primers (Table S2), PCR Master Mix (Aoko Biotechnology Co. Ltd., Beijing, China) and corresponding cycling protocol.

The mitogenome of *M. albovittata* from GenBank was used as a reference. The paired-end clean reads of *M. rufivena* and *U. puerana* were assembled in Geneious 11.0.2 (Biomatters, Auckland, New Zealand) with default parameters [19]. Geneious 11.0.2 was also used to annotate these two mitogenomic sequences. The homologous sequences of reference mitogenomes (*M. albovittata*) and open reading frames (ORFs; based on the invertebrate mitochondrial genetic code table 5) were used to predict all 13 PCGs. Then, MITOS WebServer was used to ascertain the 22 tRNA genes of each sequence based on codon table 5 [20]. The secondary structures of every tRNA were plotted with Adobe Illustrator CC2019 according to the predictions of MITOS. The 16S rRNA (*rrnL*) was located between 2 tRNA genes (*trnL1* and *trnV*), and 12S rRNA (*rrnS*) was between *trnV* and A + T control region. The A + T control region was identified using the homologous sequences of reference mitogenomes. The mitogenomic circular maps were portrayed using CGView Server [21] and perfected by Adobe Photoshop CS 6. The A + T content, AT-skews, GC-skews, nucleotide diversity (Pi value), Ka/KS ratios and evolutionary rate analysis of each PCG were all illustrated by GraphPad Prism 6.01 (San Diego, CA, USA).

### 2.3. Sequence Analysis

PhyloSuite v1.2.1 was used to calculate and analyze the nucleotide composition, codon usage of PCGs, relative synonymous codon usage (RSCU) and strand asymmetry of 13 PCGs in mitogenomes [22]. The strand asymmetry was measured by AT-skew and GC-skew with the following formulas: AT-skew = (A − T)/(A + T) and GC-skew = (G − C)/(G + C) [23]. The tandem repeat units of the A + T-control region were analysed using Tandem Repeats Finder online server (http://tandem.bu.edu/trf/trf.html) (accessed on 10 April 2021) [24]. The sliding window analysis (a sliding window of 200 bp in 20 bp overlapping steps) within DnaSP v6 was employed to estimate the Pi value of six sequences [25]. The ratios of Ka (nonsynonymous substitutions)/Ks (synonymous substitutions) based on 13 aligned protein-coding genes were estimated with DnaSP v6 [25]. MEGA X was used to estimate the mean genetic distances within six Mileewinae species under the Kimura 2-parameter mode [26]. The two complete mitogenome sequences of Mileewinae were submitted to GenBank (GenBank accession numbers: MZ326688 and MZ326689).

### 2.4. Phylogenetic Analyses

A total of 58 species of Cicadomorpha were used in phylogenetic analyses: (1) 54 species of Membracoidea; (2) 2 species of Cicadoidea (*Diceroprocta semicincta*, *Magicicada tredecula*); and (3) 2 species of Cercopoidea (*Callitettix braconoides*, *Cosmoscarta dorsimacula*) (Table 1). The 54 species of Membracoidea (50 leafhoppers and 4 treehoppers) were chosen as the ingroup, and the other 4 species (2 cicadae and 2 froghoppers) were selected as outgroups. All the mitochondrial genome sequences used in this study were downloaded from NCBI datasets. Sequences of 13 PCGs and amino acids were used to deduce the phylogenetic relationships.

The nucleotide sequences of all PCGs were extracted from PhyloSuite [22]. These sequences were then individually aligned with codon-based multiple alignments, using the MAFFT 7 and G-INS-i strategy integrated into PhyloSuite [22]. Ambiguous sites and gaps were removed from PCG alignment using GBlocks v.0.91b under the default settings [56]. Alignments of individual genes were also concatenated using PhyloSuite [22]. We removed the third codon position of the PCGs and concentrated these alignments into a large dataset. For obtaining the proper phylogenetic analysis results, alignment of individual genes was concatenated as three datasets: (1) the PCG123 matrix, including all three codon positions of the 13 PCGs (total of 10785 bp); (2) the PCG12 matrix, including the first and second codon positions of 13 PCGs (total of 7190 bp); and (3) the AA matrix, including amino acid sequences of the 13 PCGs (total of 3336 amino acids). The best-fit partitioning strategies and nucleotide substitution model for these three datasets were selected using PartitionFinder 2.2.1 using the linked branch lengths, greedy algorithm and Bayesian information criterion (BIC) model [22,57]. The results are shown in Table S3 and used for analyses of maximum likelihood (ML) and Bayesian inference (BI).

ML trees were performed using IQ-TREE under ultrafast bootstrap (UFB), and the values of Bootstrap support (BS) were evaluated with 1000 replicates [22,58]. Bayesian analysis was performed using MrBayes 3.2.6 [59]. Two simultaneous Markov chain Monte Carlo (MCMC) were run. Each MrBayes analysis of both the PCG123 matrix and PCG12 matrix involved 35,000,000 generations with sampling every 1000 generations. The analysis of the AA matrix was performed by running 5,000,000 generations with sampling every 1000 generations. The first 25% samples were abandoned as burn-in, while the remaining were used to produce a consensus tree and calculate posterior probabilities (PP). The convergence of the individual runs is indicated by the average standard deviation of split frequencies <0.01 in MrBayes 3.2.6 and effective sample size (ESS) >200 in Tracer [59,60]. All phylogenetic analyses were processed on the CIPRES Science Gateway (www.phylo.org, (accessed on 10 April 2021)) [61].

## 3. Results and Discussion

### 3.1. Mitogenome Organization and Base Composition

**Figure 1:**
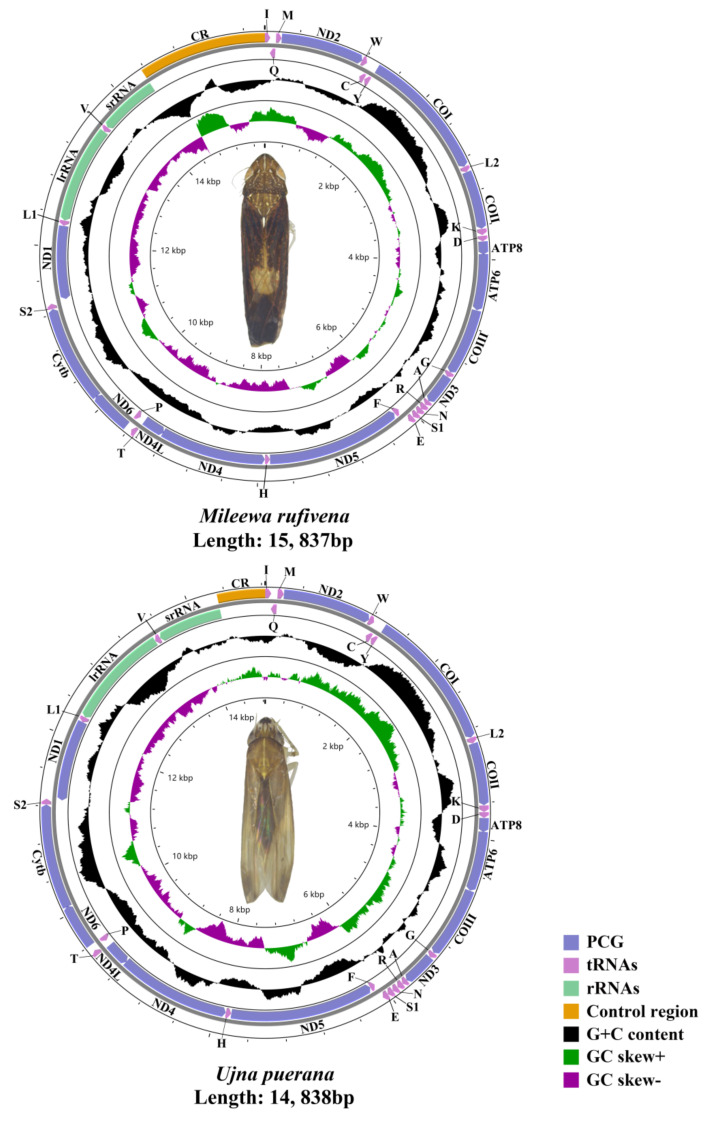
Circular maps of the mitogenome of *Mileewa rufivena* and *Ujna puerana*.

The circular map (Figure 1) was presented to visualise the mitogenomes of Mileewinae clearly. The complete mitogenomes of *M. rufivena* and *U. puerana* are circular, closed and double-stranded molecules, just as most leafhopper mitogenomes. Each mitogenome contained 13 protein-coding genes (PCGs), 22 transfer RNAs (tRNAs), two ribosomal RNA genes (rRNAs), and a putative control region (CR) (Figure 1). There are two strands on the mitochondrial genome: the majority strand (J-strand) and the minority strand (N-strand). The J-strand generally contains 23 genes (nine PCGs, 14 tRNAs) and CR, and the N-strand includes 14 genes (four PCGs, eight tRNAs and two rRNA). Gene arrangement was identical and conserved among six sequences, which is the same as the putative ancestral pattern of insects [4,62]. The length of *M. rufivena* and *U. puerana* were 15,837 bp and 14,838 bp, respectively, while these genomes of Mileewinae range in length from 14,838 bp (*U. puerana*) to 16,020 bp (*M. alara* (partial genome)) mainly due to the variable length of the A + T-control region (Table S4). The A + T-control region, general to the mitochondria of animals, might relate to the med origin of replication and promoters for transcription initiation [2].

**Figure 2:**
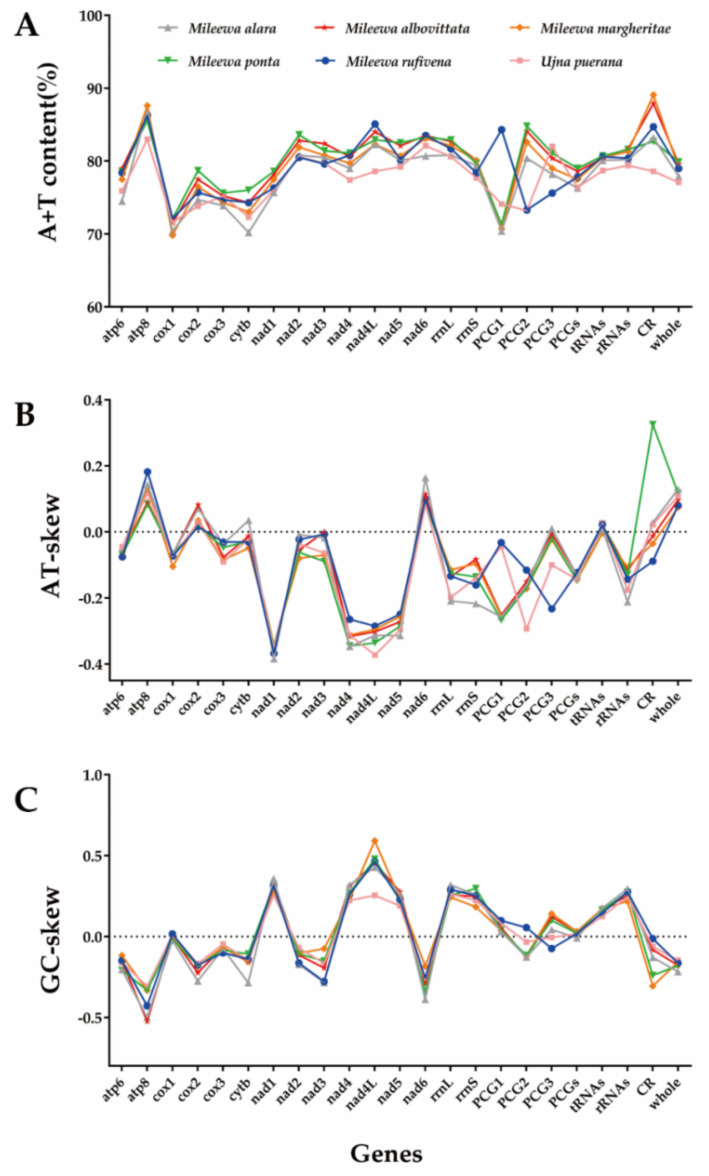
Comparison of the A + T contents, nucleotide skewness of six species of Mileewinae. (**A**) A + T content, (**B**) AT-skew and (**C**) GC-skew.

The nucleotide compositions of *M. rufivena* and *U. puerana* are shown in Table S4. Obviously, the content of adenine deoxyribonucleotide (A) and thymine deoxyribonucleotide (T) occupy a very large proportion of the entire sequence: 79.0% for *M. rufivena* and 77.1% for *U. puerana*. This intense base composition bias has also been shown in the other four Mileewinae species, which is common in insect mitogenomes [2]. The high A + T content of the whole-mitogenomes is due to the base composition of PCGs, tRNA, rRNA and the CR (Figure 2A). In the four genes, the highest A + T contents were found mainly in the CR, and *M. margheritae* had the strongest A + T bias (89.1%) compared with the other species of Mileewinae. The A + T content is similar in rRNAs and tRNA in all six species, which is usually slightly higher in rRNAs than in tRNAs. Biased A + T content was also found in the pattern of codon usage. Three codon positions of PCGs in six Mileewinae species had a comparatively close A + T content. The nucleotide skew statistics show slightly negative AT-skews and positive GC-skews in all six whole mitogenomes. Except for the slightly positive tRNAs, other regions have a moderately positive or negative AT-skew. The PCGs had slightly positive or negative GC-skews, while the value for CR is irregular; tRNAs and rRNAs have moderately positive GC-skews (Figure 2B,C).

### 3.2. Protein-Coding Genes and Codon Usage

The total length of the 13 PCGs was 10936 bp for *M. rufivena* and 10,953 bp for *U. puerana*. Of all 13 PCGs, 4 (*ND1*, *ND4*, *ND4L* and *ND5*) were encoded by N-strand and the remaining 9 (*COX1*, *COX2*, *COX3*, *ATP6*, *ATP8*, *ND2*, *ND3*, *ND6* and *Cytb*) were transcribed from the J-strand. The lengths of each PCG ranged from 153 bp (*ATP8*) to 1674 bp (*ND5*) in *Mileewa rufivena* and 153 bp (*ATP8*) to 1675 bp (*ND5*) in *Ujna puerana*. The lengths of PCGs were also highly similar in size across all six mileewine mitogenomes. These two species show a negative AT-skew (−0.123, −0.144) and a positive GC-skew (0.018, 0.016) in PCGs (Table S4 and Figure 2B,C). In the mitogenomes of *M. rufivena*, the A + T content of the first codon (84.3%) was much higher than in the second (73.3%) and in the third (75.6%). While in *U.* *puerana*, the third codon (82.0%) was much higher in A + T content than the first (74.1%) and the second (73.2%). However, the other four mileewines had a higher A + T content in the second codon than the other two (Table S4 and Figure 2A).

In the two newly sequenced mitogenomes, most of PCGs start with the typical codon ATN (ATA, ATT, ATG and ATC), whereas *ND5* and *ATP8* in *M.* *rufivena* began with a TTG codon. These abnormal initiation codons of *ND5* and *ATP8* are also reported in all other species of *Mileewa,* and this is a distinction between *Mileewa* and *Ujna* [14,15,16,17]. Three kinds of putative termination codon exist on two new mitogenomic sequences: TAG, TAA and a single T-. In *M.* *rufivena*, the incomplete stop codon T- was used in *COX2*, while the completed codon TAG was used for *ND2*, and TAA for the remaining 10 PCGs. In the *U.* *puerana* mitogenomes, codon T- was used in *COX2*, *COX3* and *ND5*; TAG was used for *ND3*; and TAA functioned for the others. TAG was not found in *M. alara* and *M. albovittata*. Meanwhile, every mitogenome sequence had the incomplete stop codon T-, and this condition is common among leafhoppers. During the process of mRNA maturation, this incomplete T- might be completed by post-transcriptional polyadenylation [63].

**Figure 3:**
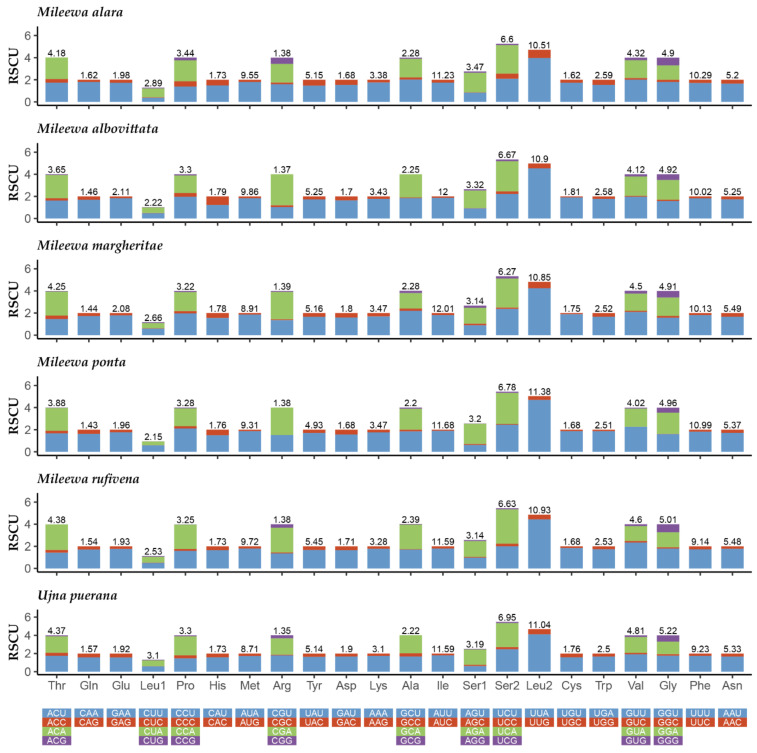
Relative synonymous codon usage (RSCU) in the mitogenomes of six Mileewinae species.

Except for the termination codon, the total number of codons is 3645 (*M. rufivena*) and 3652 (*U. puerana*). The three most abundant amino acids are Leu (*M. rufivena*: 13.46%, *U. puerana*: 14.14%), Ile (11.59%, 11.59%) and Ser (9.77%, 10.14%). Arg (1.38%, 1.35%), Glu (1.54%, 1.57%) and Cys (1.68%, 1.76%) are the least used in the two Mileewinae mitogenomes. The relative synonymous codon usage (RSCU) of mitogenomes of the six Mileewinae species is shown in Figure 3. The six amino acid codons in the largest numbers are AUU (Ile), UUA (Leu2), UUU (Phe), AUA (Met), AAU (Asn) and UAU (Tyr), which are all composed of A and U. Additionally, the three positions of each amino acid codon are more likely to use A/T than G/C (Figure 2A), reflecting the nucleotide A + T bias in the PCGs of Cicadellidae. Among 62 available codons, *M. rufivena* loses the codon Thr (ACG) and Ala (GCG) is missing in *U. puerana*.

### 3.3. Gene Overlaps and Intergenic Spacers

*M. rufivena* had 10 gene overlaps with sizes from 1 to 8 bp (Table S5), while *U. puerana* had 13 with sizes from 1 to 10 bp (Table S6). The the lengths of overlapping regions were 51 bp and 41 bp, respectively. The longest overlap regions was 8 bp between the *trnW-trnC* and *ND6-Cytb* (UCN) junction in *M. rufivena* and 10 bp between *trnS2* (UCN)*-ND1* in *U. puerana*. Likewise, the longest overlap within the other four *Mileewa* mitogenomes (*M. alara*, *M. albovittata*, *M. margheritae* and *M. ponta*) was between *trnS2* (UCN)-*ND1* (10 bp), *trnS2* (UCN)-*ND1* (10 bp), *ND6-Cytb* and *trnW-trnC* (8 bp), and *ND6-Cytb* and *trnW-trnC* (8 bp), respectively. All six Mileewinae species had the two same overlaps: *trnI-trnQ* (3 bp; TTG) and *ND6-Cytb* (8 bp; ATGAATAA).

For intergenic spacers, *M. rufivena* had 13 while *U. puerana* had 8 (Tables S5 and S6). These non-coding regions ranged from 1 to 17 bp and had a total length of 49 and 14 bp. *M. rufivena* had an unconventional intergenic spacer of 17 bp, which could not be found in other mileewine species. Meanwhile, other spacers were also quite different in that only two short gaps were shared in all six mitogenomes of Mileewinae (*trnP*-*ND6*, 2 bp; *COX2*-*trnK*, 1 bp). Overlaps were more variable, longer than intergenic spacers in this group and occurred more frequently between tRNA and tRNA, which may related to the fewer evolutionary constraints of tRNA genes [64].

### 3.4. Transfer and Ribosomal RNA Genes

**Figure 4:**
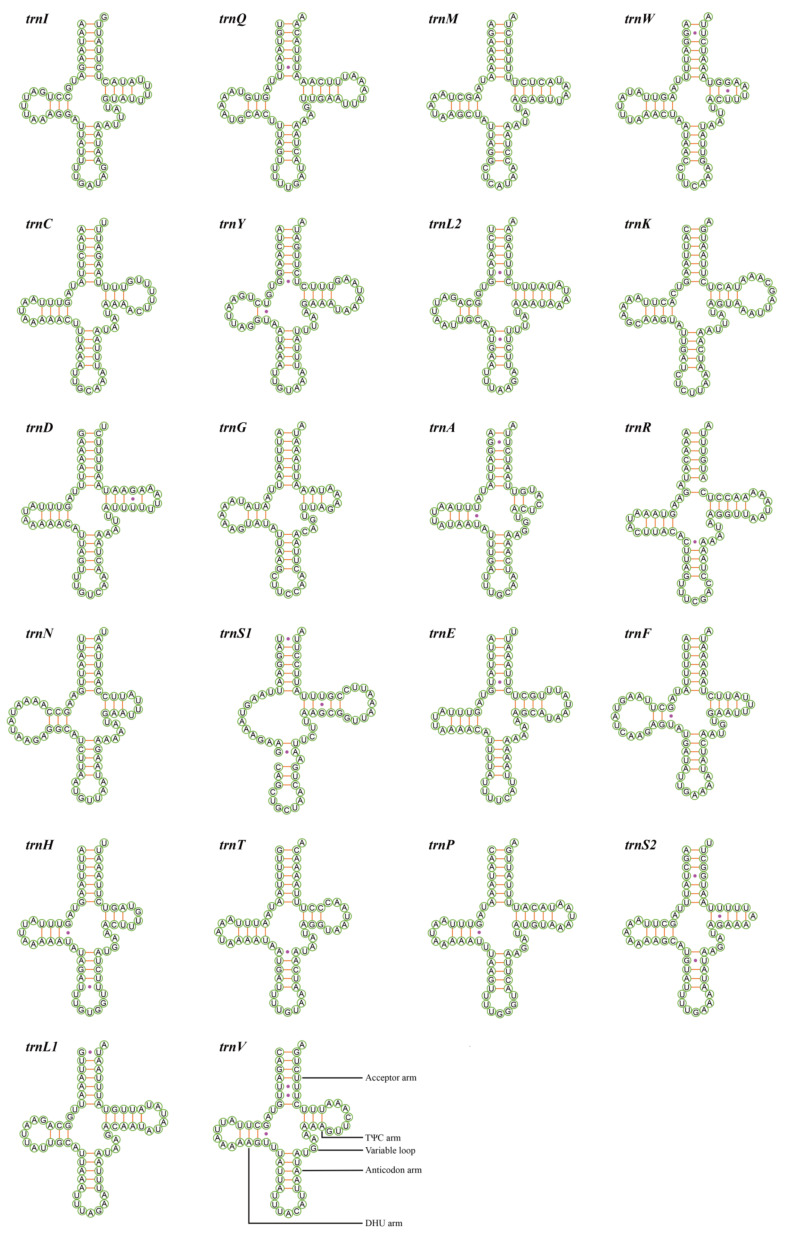
Putative secondary structure for the 22 tRNAs of *Mileewa rufivena*.

**Figure 5:**
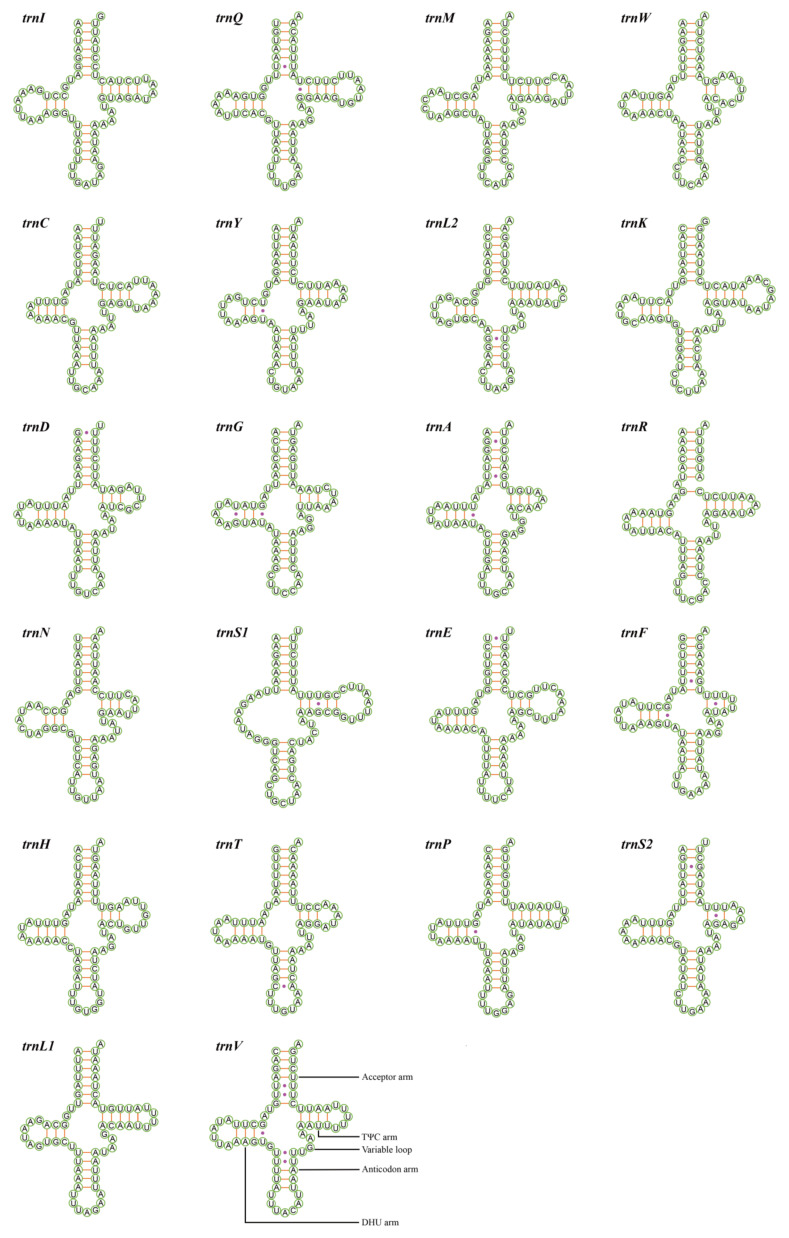
Putative secondary structure for the 22 tRNAs of *Ujna puerana*.

There are 22 typical tRNA genes interspersed in the mitogenomes of *M. rufivena* and *U. puerana*, conservatively and discontinuously. Except for four species of Cicadellidae, which had tRNA rearrangements, all positions of these 22 tRNA genes were identified in previously sequenced mitogenomes (Table 1) [22,38,39,45]. These tRNAs ranged in length from 59 to 72 bp, with a total size of 1458 bp and 1430 bp, respectively. 14 tRNAs were located on the J-strand, and the remaining eight on the N-strand (Figure 1). Putative secondary structure for the 22 tRNAs of *M. rufivena* and *U. puerana* are shown in Figure 4 and Figure 5. Except *trnS1* (AGN) with a reduced dihydrouridine (DHU) arm and with a form of loop instead, all the other 21 tRNAs could folded into the typical clover secondary structure. Compared with the previously published leafhopper and other insect mitogenomes, this kind of situation is very common [4]. Previous studies have found this dihydrouridine arm replacement loop may occur at a very early time with regard to metazoan evolution [65]. Among these six mileewines, the size of the acceptor arm (7 ntp), anticodon arm (5 ntp) and anticodon loop (7 bp) were strictly conserved, while other components were slightly variable. The anticodons were also identical and highly conserved in current mitogenomes of leafhoppers aside from five species of leafhopper (two species of Evacanthinae, one species of Deltocephalinae and two species of Megophthalminae), which employed TCT as the anticodon for *trnS1* [66,67]. Five types of missing pairings appeared in the tRNAs: UU, UG, CA, AA and a single A. There were 14 UG, 10 UU, one CA, one AA and two extra single As in *M. rufivena* and 13 UG, nine UU, one CA and one extra single A in *U. puerana*. Such non-canonical UG pairs exist in other leafhoppers as well. Beyond that, positive AT-skew and GC-skew are exhibited within the tRNAs of two new sequences (Table S4 and Figure 2B,C).

Two rRNA genes (*rrnL* and *rrnS*) of *M. rufivena* and *U. puerana* were all encoded on the N-strand. The large one (*rrnL*), between *trnL1* and *trnV*, had a length of 1218 bp and 1216 bp, respectively. While the small rRNA (*rrnS*), located between *trnV* and A + T rich regions, had lengths of 754 bp and 763 bp. These two rRNAs had a heavy AT nucleotide bias, which reached 80.4% and 79.4%, respectively. Similarly, the negative AT-skew and positive GC-skew is shown in the rRNAs of these two newly sequenced mitochondrial genomes (Table S4 and Figure 2B,C). The percentages of pairwise identity in *rrnS* and *rrnL* of the six Mileewinae species are 79.6% and 79.4%, respectively, (MAFFT alignment).

### 3.5. Control Region

The putative control region, also called the A + T rich region, was the longest non-coding region in these mitogenomes. This region is located between *rrnS* and *trnI,* and the size of this region is variable, ranging from 518 bp (*U. puerana*) to 1610 bp (*M. ponta*). The AT content is 84.7% in *M. rufivena* and 78.6% in *U. puerana*. They all have a mild positive or negative AT-skew and GC-skew (Table S4 and Figure 2B,C).

**Figure 6:**
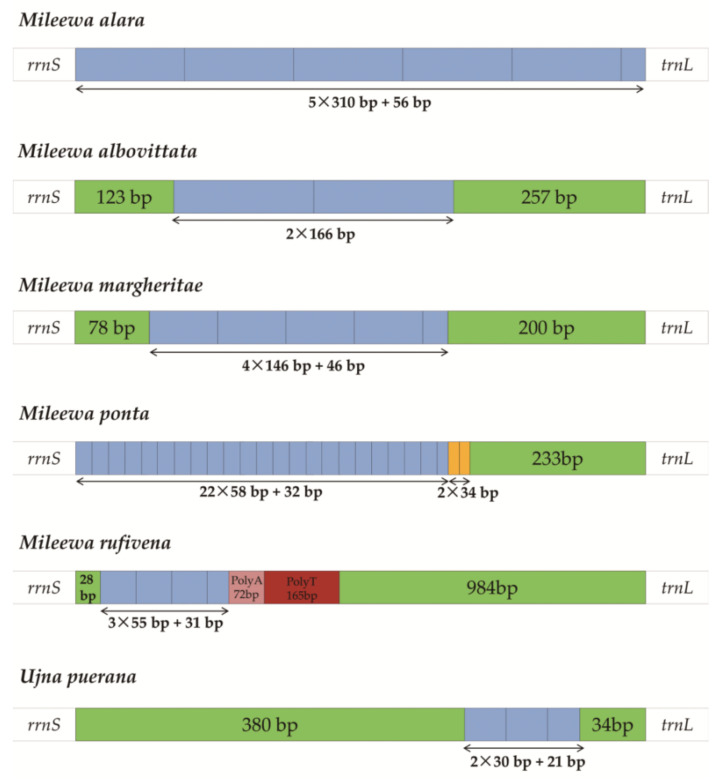
Structures of the A + T-control region in Mileewinae mitochondrial genomes. The blue and orange blocks indicate the tandem repeats, while the pink and red blocks represent the A/T repeat regions. The remaining regions are shown with green boxes.

These regions have several regulatory elements that may have a significant function in the origination of replication and transcription [2,4]. The repeat sequences vary in different mitogenomes (Figure 6). *M. alara* had the longest tandem repeat units with a size of 310 bp, while the shortest is in *U. puerana* at only 30 bp. *M. ponta* has 22 repeating units of 58 bp and another two repeat tandem units of 34 bp. The other three Mileewinae species have a relatively long size, ranging from 55 bp to 166 bp. In addition, Poly A/T stretches were only found in *M. rufivena*. The length and number of repeat units in Mileewinae are different, and we could not find any connection among them. More mitogenomes could be sequenced or more advanced methods may become available to figure out how to resolve this issue in the future.

### 3.6. Nucleotide Diversity and Evolutionary Rate Analysis

**Figure 7:**
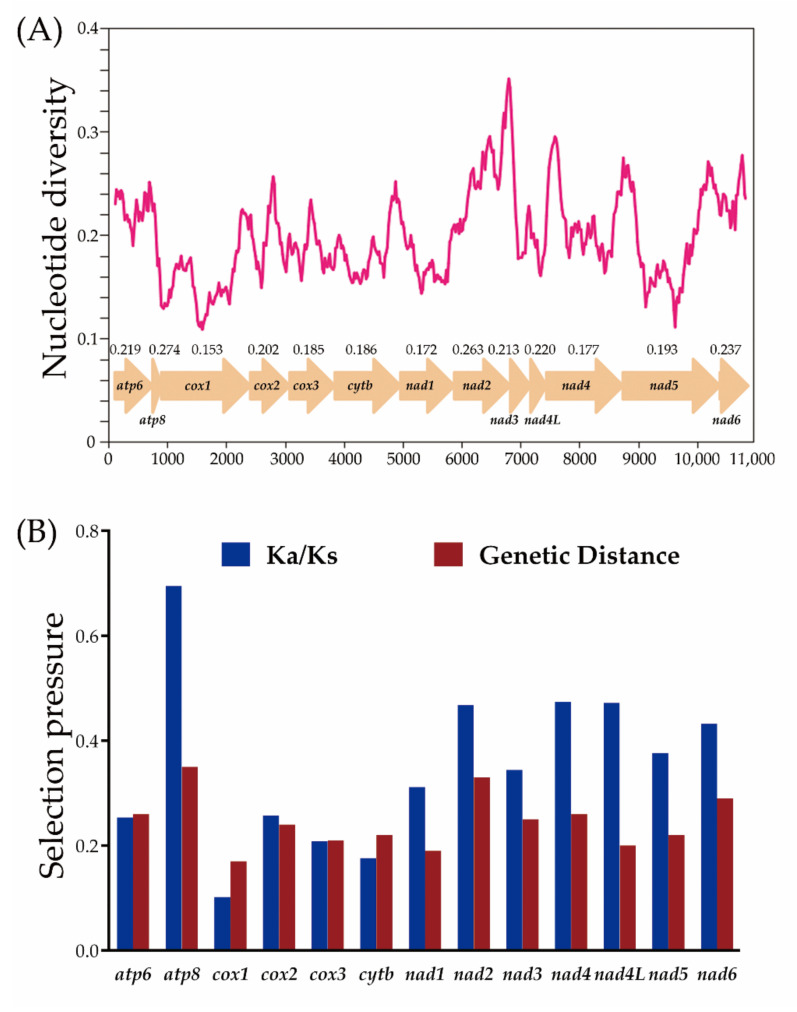
Nucleotide diversity and selection pressures on 13 PCGs in Mileewinae. (**A**) Sliding window analysis of 13 protein-coding genes among six Mileewinae species. The red curve shows the value of Pi (nucleotide diversity). Pi value of each PCG is shown above the arrows. (**B**) Genetic distances (on average) and ratio of non-synonymous (Ka) to synonymous (Ks) substitution rates of each protein-coding gene among six Mileewinae species.

The nucleotide diversity of the 13 PCGs genes among our six mileewines is displayed in Figure 7A. The four with the distinctly highest variability were *ATP8* (Pi = 0.274), *ND2* (Pi = 0.263), *ND6* (Pi = 0.237) and *ND4* (Pi = 0.220), while *COX1* (Pi = 0.153), *ND1* (Pi = 0.172), *ND4L* (Pi = 0.177) and *COX3* (Pi = 0.185) exhibited relatively low Pi values.

Genetic distance and Ka/Ks analyses also present the same trend (Figure 7B). The mean value of genetic distances within six mitogenomes shows that *ATP8* (mean value = 0.345), *ND2* (0.328) and *ND6* (0.285) have undergone a relatively fast evolution. Inversely, *COX1* (0.171), *ND1* (0.195) and *ND4L* (0.204) with lower distances are evolving comparative slowly. The pairwise Ka/Ks analyses indicate that the values of the Ka/Ks ratio (ω) of 13 PCGs range from 0.102 to 0.695 (0 < ω < 1). This indicates that these 13 genes are under a purifying selection; therefore, they are suitable for investigating phylogenetic relationships within the Cicadomorpha. *COX1*, with the lowest value of ω, experienced the strongest purifying selection, and *ATP8*, with the maximum value of 0.726, underwent weaker purifying selection. These two genes also exhibited the lowest and highest evolutionary rates, respectively. In our study, we chose *COX1* as the criteria for identifying species due to it showing the lowest variation and evolution. Therefore, this fragment gene could also be used for the taxa with close, ambiguous and highly variable morphological characters [68]. Moreover, it has long been regarded as the universal barcode for species identification.

### 3.7. Phylogenetic Relationships

**Figure 8:**
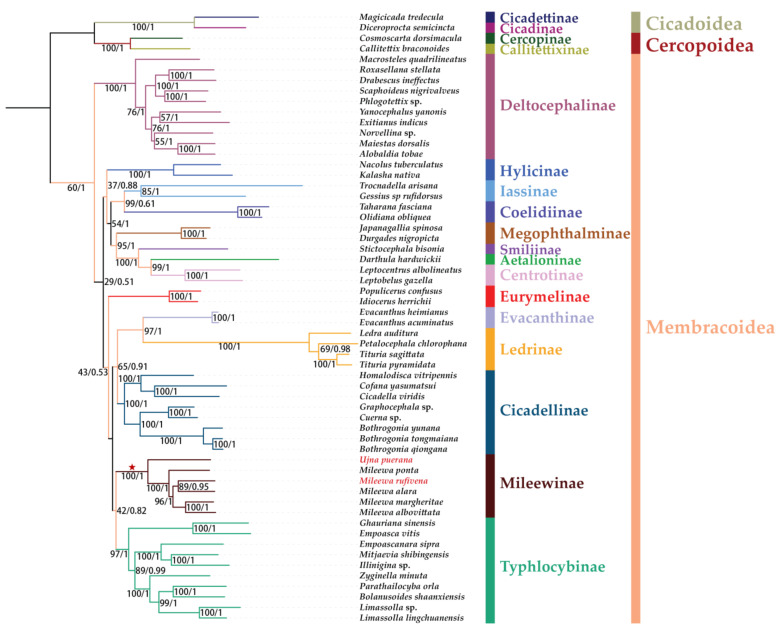
Phylogenetic tree inferred from ML and BI method based on PCG123 dataset. Supports at nodes are bootstrap support values (BS) and posterior probabilities (PP).

**Figure 9:**
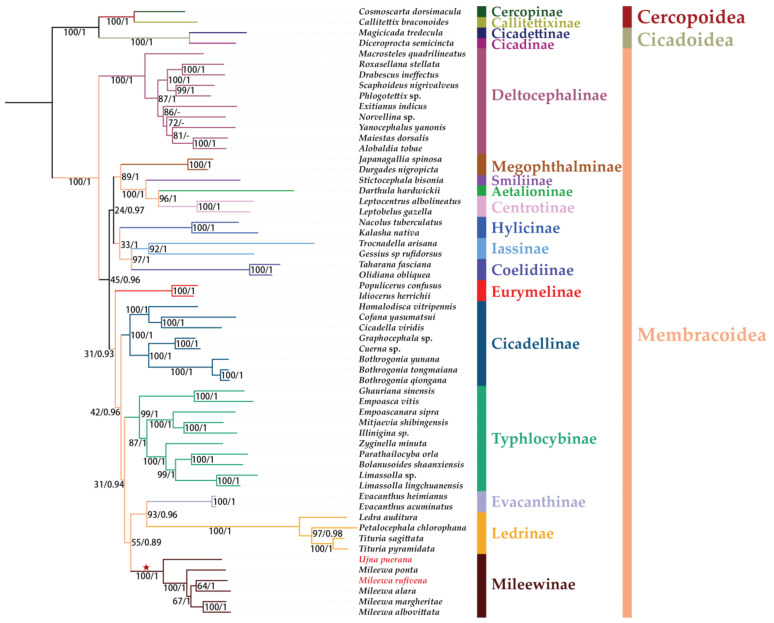
Phylogenetic tree inferred from the ML method based on PCG12 dataset and the BI method based on the AA dataset. Supports at nodes are bootstrap support values (BS) and posterior probabilities (PP). “-” indicates the clades or species are different.

**Figure 10:**
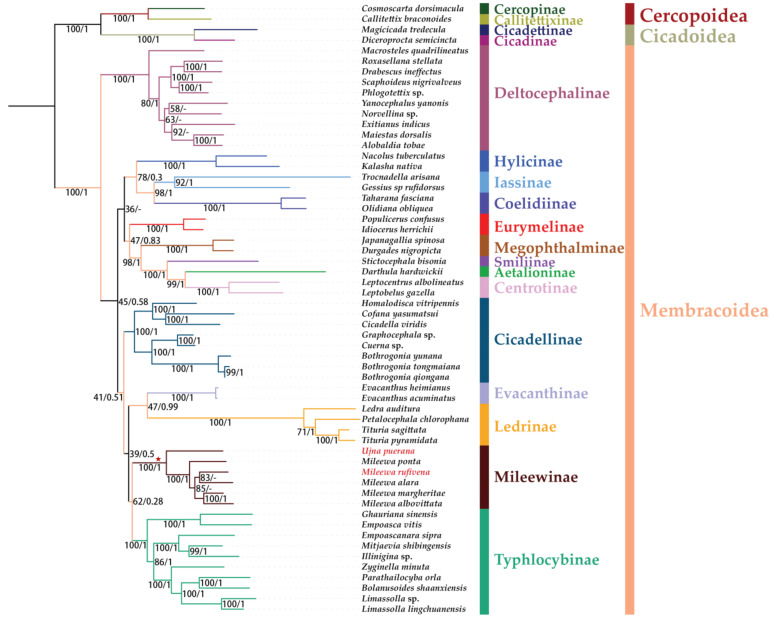
Phylogenetic tree inferred from the ML method based on AA dataset and the BI method based on the PCG12 dataset. Supports at nodes are bootstrap support values (BS) and posterior probabilities (PP). “-” indicates the clades or species are different.

Six phylogenetic trees formed from three datasets (PCG123, PCG12 and AA) were derived using two methods (ML and BI). The topological structures are exactly the same, receiving strong support in most nodes (Figure 8, Figure 9 and Figure 10). The results of the phylogenetic relationships are largely consistent with Chen et al. [69], but the species we used in our analyses were more abundant. Our putative ingroup was recovered as monophyletic with respect to Cercopoidea and Cicadoidea in all trees, with high nodal support values (bootstrap support values (BS) = 100 in ML trees and Bayesian posterior probability (PP) = 1 in BI trees). In most trees, Deltocephalinae constituted one clade as sister group to the other groups and at the base position of the tree with a strong support (BS = 100; PP = 1). Treehoppers (Aetalionidae and Membracidae) were monophyletic as a lineage flowing from leafhoppers and as sister group to Megophthalminae; this also obtained strong support (BS = 100; PP = 1). Except for Deltocephalinae being recovered as monophyletic within Cicadellidae, the relationships of the other subfamilies (Cicadellinae, Coelidiinae, Eurymelinae, Evacanthinae, Hylicinae, Iassinae, Ledrinae, Megophthalminae, Mileewinae and Typhlocybinae) varied slightly and gained lower values of BS and PP than maximum support.

Within the Cicadellidae, Mileewinae forms a monophyletic group with maximum support values in all monophyletic trees of ML and BI (BS = 100; PP = 1), which was congruent with former studies based on the *28S* sequences and mitogenomes of Mileewinae [14,15,16,17,70]. Under a disparate matrix and methods, the phylogenetic relationships between Mileewinae and other subfamilies are different: (1) ((Typhlocybinae + Mileewinae) + (Cicadellinae + (Ledrinae + Evacanthinae))) was formed in the ML/BI tree based on PCG123; (2) (((Typhlocybinae + Mileewinae) + (Ledrinae + Evacanthinae)) + Cicadellinae) was yielded by P12-BI and AA-ML; and (3) ((Mileewinae + (Ledrinae + Evacanthinae) + Typhlocybinae) + Cicadellinae) was formed in P12-ML and AA-BI. The relationships among Mileewinae, Typhlocybinae, Ledrinae and Evacanthinae are inconsistent and the support values of the branch with Mileewinae are low (BS < 62, PP < 0.89). Mileewinae could form a sister group to Typhlocybinae in most results of phylogenetic analyses and is similar to the morphological phylogeny research by Dietrich [71]. However, a different relationship, Mileewinae, Ledrinae + Evacanthinae and Typhlocybinae, forms a monophyletic group that is first found in mitogenome analysis. Nevertheless, all results exhibited here indicate that Mileewinae is a monophyly with a closer phylogenetic relationship with Typhlocybinae compared to the Cicadellinae. Meanwhile, our study is different from previous studies, which considered Mileewini as a tribe of Cicadellinae or transferred it into Typhlocybinae [9,10,12]. However, we only used one tribe in our phylogenetic analyses and the quantity of our sample is too small to be representative. Therefore, more data on mitogenomes for Mileewinae are required to confirm the monophyly of this subfamily. More sequences of leafhoppers are also needed to further test the relationships between Mileewinae and other subfamilies.

Within Mileewinae, six species (*M. ponta*, *M. rufivena*, *M.* *alara*, *M. albovittata*, *M. margheritae* and *U. puerana*) represent two genera (*Mileewa* and *Ujna*) of one tribe (Mileewini). *Mileewa* forms a sister group to *Ujna* in all phylogenetic trees with strong support (BS = 100; PP = 1). Six Mileewini species could be recovered with the topology (*U. puerana* + (*M. ponta* + (*M. rufivena* + *M. alara*) + (*M. albovittata* + *M. margheritae*))) in a phylogenetic tree based on PCG123-ML, PCG123-ML, PCG12-ML, AA-ML and AA-BI, with middle to high support. However, only one sampling of *Ujna* is employed in our analysis, so more mitochondrial data concerning this tribe may be added to analyze the internal structure of this group in future work.

## 4. Conclusions

In this study, two new, complete mitogenomes (*Mileewa rufivena* and *Ujna puerana*) have been sequenced and have quite similar features in the size of each genome, base content, AT nucleotide bias, AT–skew, GC–skew, codon usage of protein genes and secondary structure of tRNA. Their gene arrangement is identical and conserved with alignment to the putative ancestral pattern of insects. All protein-coding genes of *U*. *puerana* began with the start codon ATN, while five *Mileewa* species had an abnormal initiation codon TTG in *ND5* and *ATP8*. Moreover, *M. rufivena* had an intergenic spacer of 17 bp that could not be found in other mileewine species.

Phylogenetic analysis is based on three datasets (PCG123, PCG12 and AA) with two methods (maximum likelihood and Bayesian inference), and recovered the Mileewinae as a monophyletic group with strong support values. All results indicate Mileewinae has a closer phylogenetic relationship to Typhlocybinae compared to Cicadellinae. Additionally, six species within Mileewini showed the relationship (*U. puerana* + (*M. ponta* + (*M. rufivena* + *M. alara*) + (*M. albovittata* + *M. margheritae*))) in most of the phylogenetic trees generated. These results offer a valuable framework to Cicadellidae and could ultimately contribute to understanding the taxonomic status and phylogenetic relationships of Mileewinae. More mitogenomic data for Mileewinae should be added to verify the monophyly of Mileewinae and elucidate the relationships between Mileewinae and other subfamilies and define the internal structure of this group.

## Figures and Tables

**Table 1:** List of mitogenomes used for phylogenetic analysis.

Superfamily	Family	Subfamily	Species	Accession Number	Reference
Cicadoidea	Cicadidae	Cicadinae	*Diceroprocta semicincta*	KM000131	Unpublished
		Cicadettinae	*Magicicada tredecula*	MH937705	[27]
Cercopoidea	Cercopidae	Callitettixinae	*Callitettix braconoides*	NC_025497	[28]
		Cercopinae	*Cosmoscarta dorsimacula*	NC_040115	Unpublished
Membracoidea	Aetalionidae	Aetalioninae	*Darthula hardwickii*	NC_026699	[29]
	Membracidae	Centrotinae	*Leptobelus gazella*	JF801955	[30]
			*Leptocentrus albolineatus*	NC_044707	[30]
		Smiliinae	*Stictocephala bisonia*	MW342606	[31]
	Cicadellidae	Cicadellinae	*Bothrogonia qiongana*	NC_049894	Unpublished
			*Bothrogonia tongmaiana*	NC_049895	Unpublished
			*Bothrogonia yunana*	NC_049896	Unpublished
			*Cicadella viridis*	KY752061	Unpublished
			*Cofana yasumatsui*	NC_049087	[32]
			*Cuerna* sp.	KX437741	[33]
			*Graphocephala* sp.	KX437740	[33]
			*Homalodisca vitripennis*	NC_006899	Unpublished
		Coelidiinae	*Olidiana obliquea*	MN780583	[34]
			*Taharana fasciana*	NC_036015	[35]
		Deltocephalinae	*Alobaldia tobae*	KY039116	[36]
			*Drabescus ineffectus*	NC_050258	[37]
			*Exitianus indicus*	KY039128	[35]
			*Macrosteles quadrilineatus*	NC_034781	[38]
			*Maiestas dorsalis*	NC_036296	[39]
			*Norvellina* sp.	KY039131	[36]
			*Phlogotettix* sp.	KY039135	[36]
			*Roxasellana stellata*	NC_050257	[37]
			*Scaphoideus nigrivalveus*	KY817244	[40]
			*Yanocephalus yanonis*	NC_036131	[36]
		Eurymelinae	*Idiocerus herrichii*	MN935487	[41]
			*Populicerus confusus*	NC_050982	Unpublished
		Evacanthinae	*Evacanthus acuminatus*	MK948205	[42]
			*Evacanthus heimianus*	MG813486	[43]
		Hylicinae	*Kalasha nativa*	MW218662	[44]
			*Nacolus tuberculatus*	MW218663	[44]
		Iassinae	*Gessius rufidorsus*	MN577633	[45]
			*Trocnadella arisana*	NC_036480	[45]
		Ledrinae	*Ledra auditura*	MK387845	[46]
			*Petalocephala chlorophana*	NC_051527	[47]
			*Tituria pyramidata*	NC_046701	Unpublished
			*Tituria sagittata*	NC_051528	[47]
		Megophthalminae	*Durgades nigropicta*	NC_035684	[48]
			*Japanagallia spinosa*	NC_035685	[48]
		Mileewinae	*Mileewa alara*	MW533151	[17]
			*Mileewa albovittata*	MK138358	[14]
			*Mileewa margheritae*	MT483998	[16]
			*Mileewa ponta*	MT497465	[15]
			*Mileewa rufivena*	MZ326689	This study
			*Ujna puerana*	MZ326688	This study
		Typhlocybinae	*Bolanusoides shaanxiensis*	MN661136	Unpublished
			*Empoasca vitis*	NC_024838	[49]
			*Empoascanara sipra*	NC_048516	[50]
			*Ghauriana sinensis*	MN699874	[51]
			*Illinigina* sp.	KY039129	[36]
			*Limassolla lingchuanensis*	NC_046037	[52]
			*Limassolla* sp.	MT683892	[53]
			*Mitjaevia shibingensis*	MT981879	Unpublished
			*Parathailocyba orla*	MN894531	[54]
			*Zyginella minuta*	NC_052876	[55]

## Data Availability

Date available on request.

## Supplementary Materials

The following are available online at https://www.mdpi.com/article/10.3390/insects12080668/s1, Table S1: Collection information of the Mileewinae species in this study. Table S2: Primers used for mitogenome analysis. Table S3. Best partitioning scheme and nucleotide substitution models for different datasets selected by PartitionFinder. Table S4: Nucleotide composition and skewness comparison of Mileewinae mitogenomes. Table S5: Mitogenomic organization of *Mileewa rufivena*. Table S6: Mitogenomic organization of *Ujna puerana*.
