# Supplementary material for: Two Complete Mitochondrial Genomes of Mileewinae (Hemiptera: Cicadellidae) and a Phylogenetic Analysis
Source: Insects. 2021 Jul 22;12(8):668. doi: 10.3390/insects12080668 (PMC8396625; doi:10.3390/insects12080668)
Supplement: Supplementary file 1 [file insects-12-00668-s001.zip › insects-1271424-supplementary.pdf]

Supplementary Material

Two Complete Mitochondrial Genomes of Mileewinae (Hemiptera: Cicadellidae) and a Phylogenetic Analysis

Tinghao Yu and Yalin Zhang *

Key Laboratory of Plant Protection Resources and Pest Management, Ministry of Education, Entomological Museum, College of Plant Protection, Northwest A&F University, Yangling, Shaanxi 712100, China; 18404965218@163.com (T.Y.)
* Correspondence: yalinzh@nwsuaf.edu.cn; Tel.: +86-029-87092190

Table S1. Collection information for the Mileewinae species in this study.

Name	Locality	Time	Collector
Mileewa rufivena	Mountain Fanjing, Guizhou, China	30-April-2018	Lin-Shuanghu
Ujna puerana	Five Finger Mountain, Hainan, China	8-July-2018	Du-Lan

Table S2. Primers used for mitogenome analysis. F: forward; R: reverse.

No.	Primer sequence (5'-3')	Annealing temperature	Amplified region
1	F: AATTAAGCTACTAGGTTCATACCC	52 °C	ND2 (980 bp)
	R: ACARCTTTGAAGGYTAWTAGTTT	52 °C	
2	F:GCCTGATAAAAAGGRTTAYYTTGATA	51 °C	trnM-ND2-trnW (1560 bp)
	R: TTTATTCGTGGRAATGCYATRTC	51 °C	

Table S3. Best partitioning scheme and nucleotide substitution models for different datasets selected by PartitionFinder.

Data Matrix	Subset Partitions	Model
PCG123-BI	1. *nad6*_pos1, *atp6*_pos1	GTR + I + G
	2. *cox3*_pos2, *atp6*_pos2, *cox2*_pos2, *cytb*_pos2	GTR + I + G
	3. *cytb*_pos3, *atp6*_pos3, *nad6*_pos3, *atp8*_pos3, *nad3*_pos3,	GTR + G
	4. *atp8*_pos1, *nad3*_pos1, *nad2*_pos1	GTR + I + G
	5. *atp8*_pos2, *nad2*_pos2, *nad6*_pos2, *nad3*_pos2	GTR + I + G
	6. *cox1*_pos1	GTR + I + G
	7. *cox1*_pos2	GTR + I + G
	8. *cox1*_pos3, *cox3*_pos3, *cox2*_pos3	HKY + G
	9. *cytb*_pos1, *cox2*_pos1, *cox3*_pos1	GTR + I + G
	10. *nad1*_pos1, *nad4L*_pos1, *nad4*_pos1, *nad5*_pos1	GTR + I + G
	11. *nad1*_pos2, *nad4L*_pos2, *nad5*_pos2, *nad4*_pos2	GTR + I + G
	12. *nad1*_pos3	HKY + G

	13. *nad2*_pos3	GTR + G
	14. *nad5*_pos3, *nad4L*_pos3, *nad4*_pos3	HKY + I + G
PCG123-ML	1. *nad6*_pos1, *atp6*_pos1	GTR + I + G
	2. *cytb*_pos2, *cox3*_pos2, *atp6*_pos2, *cox2*_pos2	TVM + I + G
	3. *cytb*_pos3, *atp8*_pos3, *atp6*_pos3, *nad3*_pos3, *nad6*_pos3	GTR + G
	4. *atp8*_pos1, *nad3*_pos1, *nad2*_pos1	TIM + I + G
	5. *atp8*_pos2, *nad2*_pos2, *nad6*_pos2, *nad3*_pos2	GTR + I + G
	6. *cox1*_pos1	GTR + I + G
	7. *cox1*_pos2	TVM + I + G
	8. *cox1*_pos3	HKY + G
	9. *nad1*_pos1, *cox3*_pos1, *cox2*_pos1	GTR + I + G
	10. *cox3*_pos3, *cox2*_pos3	HKY + G
	11. *nad1*_pos1, *nad4L*_pos1, *nad4*_pos1, *nad5*_pos1	GTR + I + G
	12. *nad1*_pos2, *nad4L*_pos2, *nad5*_pos2, *nad4*_pos2	GTR + I + G
	13. *nad1*_pos3	HKY + G
	14. *nad2*_pos3	TRN + G
	15. *nad5*_pos3, *nad4*_pos3, *nad4L*_pos3	HKY + I + G
PCG12-BI	1. *nad3*, *atp6*	GTR + I + G
	2. *atp8, nad6*	GTR + I + G
	3. *cox1*	GTR + I + G
	4. *cytb*, *cox2*, *cox3*	GTR + I + G
	5. *nad1*	GTR + I + G
	6. *nad2*	GTR + I + G
	7. *nad4, nad4L,nad5*	GTR + I + G
PCG12-ML	1. *nad3*, *apt6*	GTR + I + G
	2. *atp8, nad6*	GTR + I + G
	3. *cox1*	GTR + I + G
	4. *cytb*, *cox3*, *cox2*	GTR + I + G
	5. *nad1*	GTR + I + G
	6. *nad2*	GTR + I + G
	7. *nad4, nad5, nad4L*	TVM + I + G
AA-BI	1. *atp6*, *cox2*, *nad4*, *nad5*	MTREV + I + G
	2. *nad4L, nad3, atp8, nad1*	MTREV + I + G
	3. *cox1*	MTREV+ I + G
	4. *cox3*, *cytb*	MTREV + I + G
	5. *nad6*, *nad2*	MTREV + I + G
AA-ML	1. *atp8*, *nad3, nad6, cox2, cytb, atp6, nad2, cox3*	MTART + I + G + F
	2. *cox1*	MTART + I + G
	3. *nad4L*, *nad5*, *nad1*, *nad4*	MTART + I + G + F

Table S4. Nucleotide composition and skewness comparison of Mileewinae mtgenomes.

Species	Feature	Size(bp)	T%	C%	A%	G%	A+ T%	AT-skew	GC-skew
M. alara	PCGs	10964	43.2	11.9	33.1	11.7	76.3	-0.132	-0.009
	Control region	1607	40.4	9.5	42.8	7.3	83.2	0.028	-0.130
	1st codon position	3655	44.2	14.4	26.2	15.3	70.4	-0.257	0.030
	2st codon position	3655	46.7	10.9	33.7	8.5	80.4	-0.162	-0.127
	3st codon position	3654	38.7	10.4	39.5	11.3	78.2	0.010	0.043
	tRNAs	1439	39.7	8.2	40.4	11.7	80.1	0.010	0.175
	rRNAs	2037	48.6	7.0	31.6	12.8	80.2	-0.212	0.294
	Whole genome	16020	33.9	13.4	44.0	8.6	77.9	0.129	-0.217
M. albovittata	PCGs	10961	44.5	10.4	34.1	11.0	78.6	-0.132	0.030
	Control region	714	44.5	6.4	43.4	5.5	87.9	-0.013	-0.082
	1st codon position	3654	44.6	13.6	26.6	15.2	71.2	-0.252	0.055
	2st codon position	3654	48.4	9.0	35.7	6.9	84.1	-0.151	-0.129
	3st codon position	3653	40.4	8.6	40.0	11	80.4	-0.005	0.122
	tRNAs	1420	39.3	8.2	41.1	11.5	80.4	0.022	0.168
	rRNAs	1963	45.6	6.8	36.1	11.5	81.7	-0.116	0.253
	Whole genome	15079	35.9	12.0	43.7	8.4	79.6	0.097	-0.178
M. margheritae	PCGs	10953	44.4	10.8	33.1	11.4	77.5	-0.146	0.030
	Control region	908	46.1	7.0	43.0	3.7	89.1	-0.036	-0.306
	1st codon position	3651	44.3	14.1	26.4	15.2	70.7	-0.254	0.038
	2st codon position	3651	48.4	9.4	34.2	7.4	82.6	-0.172	-0.119
	3st codon position	3651	40.4	8.9	38.6	11.8	79.0	-0.023	0.141
	tRNAs	1440	40.4	8.0	40.1	11.3	80.5	-0.004	0.173
	rRNAs	2050	45.0	7.2	36.3	11.2	81.3	-0.107	0.217
	Whole genome	15375	36.6	12	42.4	8.7	79.0	0.074	-0.158
M. ponta	PCGs	10950	45.3	10.2	33.7	10.6	79.0	-0.147	10950
	Control region	1610	31.0	10.7	51.7	6.6	82.7	0.325	-0.240
	1st codon position	3650	45.1	13.8	26.2	14.9	71.3	-0.266	0.039
	2st codon position	3650	49.6	8.4	35.2	6.6	84.8	-0.169	-0.117
	3st codon position	3650	41.3	8.5	39.8	10.4	81.1	-0.019	0.100
	tRNAs	1440	39.4	8.0	41.3	11.1	80.7	0.023	0.164
	rRNAs	1993	46.1	6.7	35.5	11.7	81.6	-0.129	0.273
	Whole genome	15999	35.3	11.8	44.6	8.2	79.9	0.117	-0.18
M. rufivena	PCGs	10936	43.7	10.9	34.1	11.3	77.8	-0.123	0.018
	Control region	1476	46.1	7.7	38.6	7.5	84.7	-0.089	-0.013
	1st codon position	3646	43.5	7.0	40.8	8.6	84.3	-0.032	0.098
	2st codon position	3645	40.9	12.6	32.4	14.1	73.3	-0.116	0.055
	3st codon position	3645	46.6	13.1	29.0	11.3	75.6	-0.233	-0.074
	tRNAs	1445	39.4	8.3	41.2	11.1	80.6	0.022	0.146
	rRNAs	1972	46.0	7.1	34.4	12.5	80.4	-0.144	0.275
	Whole genome	15837	36.3	12.2	42.7	8.8	79.0	0.080	-0.165
U. puerana	PCGs	10956	43.7	11.6	32.7	12.0	76.4	-0.144	0.016

Control region	518	38.4	11.4	40.2	10.0	78.6	0.022	-0.063
1st codon position	3652	38.7	11.9	35.4	14.0	74.1	-0.045	0.083
2st codon position	3652	47.3	13.8	25.9	12.9	73.2	-0.293	-0.034
3st codon position	3652	45.1	9.1	36.9	9.0	82.0	-0.100	-0.006
tRNAs	1422	38.3	9.4	40.4	12.0	78.7	0.027	0.125
rRNAs	1979	46.7	7.7	32.7	12.8	79.4	-0.176	0.246
Whole genome	14838	34.4	13.1	42.7	9.8	77.1	0.108	-0.147

Table S5. Mitogenomic organization of *Mileewa rufivena*.

Name	Location		Size(bp)	Intergeni nucleotid	Codon		Strand
	From	To			Start	Stop	
trnI	1	65	65	0			J
trnQ	63	130	68	-3			N
trnM	131	196	66	0			J
nad2	197	1165	969	0	ATA	TAG	J
trnW	1164	1227	64	-2			J
trnC	1220	1285	66	-8			N
trnY	1291	1357	67	5			N
cox1	1361	2896	1536	3	ATG	TAA	J
trnL2(UUR)	2898	2963	66	1			J
cox2	2964	3642	679	0	ATA	T	J
trnK	3644	3715	72	1			J
trnD	3724	3788	65	8			J
atp8	3789	3941	153	0	TTG	TAA	J
atp6	3935	4588	654	-7	ATG	TAA	J
cox3	4591	5370	780	2	ATG	TAA	J
trnG	5370	5431	62	-1			J
nad3	5432	5785	354	0	ATT	TAA	J
trnA	5785	5843	59	-1			J
trnR	5845	5912	68	1			J
trnN	5910	5976	67	-3			J
trnS1(ACN)	5976	6041	66	-1			J
trnE	6045	6110	66	3			J
trnF	6111	6177	67	0			N
nad5	6181	7854	1674	3	TTG	TAA	N
trnH	7855	7916	62	0			N
nad4	7918	9237	1320	1	ATG	TAA	N
nad4L	9231	9506	276	-7	ATG	TAA	N
trnT	9509	9573	65	2			J
trnP	9574	9640	67	0			N
nad6	9643	10134	492	2	ATC	TAA	J
cytb	10127	11263	1137	-8	ATG	TAA	J
trnS2(UCN)	11264	11326	63	0			J
nad1	11344	12255	912	17	ATT	TAA	N
trnL1(CUN)	12256	12323	68	0			N
rrnL	12324	13541	1218	0			N
trnV	13542	13607	66	0			N
rrnS	13608	14361	754	0			N
CR	14362	15837	1476	0			J

Table S6. Mitogenomic organization of *Ujna puerana*.

Name	Location		Size(bp)	Intergenic nucleotide	Codon		Strand
	From	To			Start	Stop	
trnI	1	67	67	-3			J
trnQ	65	133	69	1			N
trnM	135	203	69	0			J
nad2	204	1169	966	-2	ATA	TAA	J
trnW	1168	1230	63	-8			J
trnC	1223	1286	64	4			N
trnY	1291	1353	63	-2			N
cox1	1352	2887	1536	1	ATG	TAA	J
trnL2(UUR)	2889	2953	65	0			J
cox2	2954	3632	679	1	ATG	T	J
trnK	3634	3705	72	0			J
trnD	3706	3768	63	0			J
atp8	3769	3921	153	-4	ATA	TAA	J
atp6	3918	4568	651	1	ATA	TAA	J
cox3	4570	5347	778	0	ATG	T	J
trnG	5348	5409	62	0			J
nad3	5410	5763	354	-2	ATA	TAG	J
trnA	5762	5821	60	-1			J
trnR	5821	5885	65	2			J
trnN	5888	5950	63	-1			J
trnS1(ACN)	5950	6015	66	-1			J
trnE	6015	6079	65	-1			J
trnF	6079	6139	61	0			N
nad5	6140	7814	1675	0	ATG	T	N
trnH	7815	7878	64	0			N
nad4	7879	9192	1314	-4	ATA	TAA	N
nad4L	9189	9464	276	2	ATG	TAA	N
trnT	9467	9529	63	0			J
trnP	9530	9594	65	2			N
nad6	9597	10088	492	-8	ATC	TAA	J
cytb	10081	11217	1137	-1	ATG	TAA	J
trnS2(UCN)	11217	11278	62	-10			J
nad1	11269	12213	945	-3	ATA	TAA	N
trnL1(CUN)	12211	12275	65	0			N
rrnL	12276	13491	1216	0			N
trnV	13492	13557	66	0			N
rrnS	13558	14320	763	0			N
CR	14321	14838	518	0			J
